# Elastography of the thyroid nodule, cut-off points between benign and malignant lesions for strain, 2D shear wave real time and point shear wave: a correlation with pathology, ACR TIRADS and Alpha Score

**DOI:** 10.3389/fendo.2023.1182557

**Published:** 2023-06-16

**Authors:** Glenn Mena, Alejandro Montalvo, Michael Ubidia, Julio Olmedo, Ana Guerrero, Jose E. Leon-Rojas

**Affiliations:** ^1^ Institute of Radiology and Interventionism, Alpha Imagen, Quito, Ecuador; ^2^ ECVIEW International, Biomedical Engineering Department, Mindray, Quito, Ecuador; ^3^ Research Department, Medignosis, Quito, Ecuador; ^4^ Medical School, Universidad de las Américas (UDLA), Quito, Ecuador

**Keywords:** thyroid elastography, thyroid nodule, 2D SWE, RT SWE, pSWE, strain elastography, TIRADS, Alpha Score

## Abstract

**Objective:**

A prospective cross-sectional investigation of 170 thyroid nodules (TN) between January 2020 and December 2021 at Alpha Imagen was conducted to determine cut-off points (C/O) for elastography measurements and their diagnostic accuracy.

**Methods:**

Nodules were categorized by ACR TI-RADS, Alpha Score (AS), and Bethesda; all were evaluated using 2D Shear Wave Real Time Elastography (RT-SWE), point Shear Wave (pSWE), and Strain Elastography (SE). Data was assessed with ROC curves, the Shapiro-Wilk test, T test, Chi-square test, and ANOVA.

**Results:**

C/O were as follows: RTSWE Emax of 115kPa and 6.5 m/s, Emean of 47.5 kPa and 4.1 m/s, pSWE (average) of 52.4 kpa and 4.15 m/s; sensitivity of 81.2% and specificity of 57.6%, with a PPV of 72.4% and NPV of 70.0%. SE Value A had a C/O of 0.20%, with a sensitivity of 84%, specificity of 57%, PPV of 72.4% and NPP of 73.6%. The Strain Ratio nodule/tissue C/O was calculated as 2.69, with a sensitivity of 84%, specificity of 57%, PPV of 72.3%, and NPV of 73.5%. The RLBIndex quality control must be at least 92%; for pSWE, we suggest a mean interquartile ratio of ≤15.7% for kPa and 8.1% for m/s. The recommended depth is between 1.2 and 1.5 cm, and commonly used ROI boxes were 3x3 and 5x5mm.

**Conclusion:**

2D-SWE and pSWE with Emax and Emean demonstrated C/O with excellent diagnostic accuracy. To maximize the correct classification of TN, we suggest combining ACR TI-RADS and AS with any of the elastography measurements assessed here.

## Introduction

Ultrasound Elastography is a technique that takes advantage of the biomechanical characteristics of the tissue, measuring such characteristics by applying an external force (i.e., compression or shear) and analyzing how the tissue changes in shape and size; these changes relate to the tissue’s stiffness (1). The commercially available ultrasound elastography methods differ from each other by how they generate this force to deform the tissue and how they display this deformation; the methods available include ***strain elastography (SE)***, ***acoustic radiation force impulse (ARFI) elastography***, and ***shear-wave elastography (SWE)*** (1, 2). When applying these techniques to thyroid nodules (TN), the premise is that these biomechanical properties will vary between benign and malignant nodules; therefore, thyroid elastography and its measurements (both qualitative and quantitative) can be used as a biomarker to differentiate malignancy in TN.

***Thyroid elastography (TE)*** is being used more frequently as a diagnostic method to differentiate TN malignancy; additionally, when using it for diffuse lesions (3), there is greater confidence due to its good correlation with predictors of malignancy such as the American College of Radiology Thyroid Imaging Reporting and Data Systems (ACR TI-RADS) (4), the American Thyroid Association (ATA) (5), or the European Thyroid Imaging-Reporting and Data System (EU TIRADS) (6). TE also performs well as a standalone tool and correlates well with the latest Bethesda classification (7).

As mentioned before, the available elastography techniques differ in the way they apply the force and the measurement or quality they display after the application of that force (1, 2). ***Strain elastography (SE)*** uses a quasi-static force usually generated by pressing with the imaging transducer or by internal forces (i.e., arterial pulsations) which generate strain and displays it; ***acoustic radiation force impulse (ARFI)***, in contrast, uses a dynamic force generated by an ultrasound radiation force impulse that causes a targeted displacement of the tissue; in both ***shear-wave elastography (SWE)*** and ***point shear-wave elastography (pSWE)*** a dynamic force is generated by an ultrasound radiation force impulse which causes shear-waves that travel across the tissue, the difference is that ***pSWE*** measures the speed of localized and transient shear-waves whereas ***SWE*** produces two- or three-dimensional quantitative images of shear-wave speed (2). We will discuss the use of these different techniques in differentiating benign and malignant TN.

Multiple studies focusing on TE have been published throughout the years, for instance studies used to employ ***SE*** using different color maps, such as the Asteria (8) and Rago (9) classifications which have been progressively discontinued because quantitative TE (***SWE*** and ***pSWE***) have shown better results. ***SE*** is used to detect local deformation of the tissue – known as strain– when applying a light force in a specific region of tissue – known as the ***region of interest*** (***ROI***) (1). These results in specific values ​​of the elastic deformation of the TN, one of which is known as ***value A***; it represents the percentage of deformation of the TN, calculated considering the complete circumference of the nodule (1). An example of the use of this measurement is the result obtained by Zhang et al, with a ***value A*** of 0.21% as the ***cut-off point (C/O)*** to differentiate benign from malignant lesions (10). Another commonly used measurement of ***SE*** is the ***Strain Ratio (SR)***, which usually represents the ratio of ***value A*** to a value obtained in a sector of normal thyroid tissue –known as ***value B–*** which represents the percentage of deformation of the healthy thyroid tissue (i.e., away from the TN) (11); this ratio is known as ***SR nodule/tissue (SR N/T).*** Several studies have been published related to this latter measurement, for example one reports a value of 2.32 with a sensitivity of 95.2% and specificity of 86.5% to differentiate malignant TN (12). On the other hand, when we calculate the ratio of ***value A*** to the strain in a ROI placed in the ***sternocleidomastoid muscle (SCM)*** (11), we obtain the ratio known as ***SR nodule/muscle (SR N/M)*,** of which reports show a ***C/O*** of 3.59 with sensitivity of 100% and specificity of 86.4% (11). Finally, the ***elasticity contrast index (ECI)***, which uses ***SE*** but takes advantage of the carotid artery pulsations as the force that compresses the thyroid tissue, has also been analyzed in studies that reported that the values ​​for malignant TN were significantly higher than benign TN (3.67 vs. 1.80), the best C/O was of 2.16, with a sensitivity of 90.3%, specificity of 82.9%, Positive Predictive Value (PPV) of 83.7% and Negative Predictive Value (NPV) of 91.2% (12).

In quantitative ***TE***, ***C/Os*** are very diverse, for example, Liao et al. published results of ROC curves with ***SWE***, reporting an ***elastography mean value (Emean)*** of 32 kPa with sensitivity, specificity, PPV, and NPV of 81%, 65%, 23%, and 96%, respectively (13). Additionally, studies have also employed new TE measurements such as the ***2D Shear Wave (2D-SWE)***, with the best reported ***C/O*** at 34.5 kPa for ***Emean*** (Sensitivity 83.7%, Specificity 77.4%, VPP 63.3% and NPV 89.7%) (14); the results of Farghadani, in contrast, report an optimal value at 39.6 kPa (15), with other studies reporting higher ***C/O*** (94 kPA) ​​with good results (16). When using ***SWE*** in units of meters/second (m/s), the best ***C/O***, reported by Aghaghazvini L et al, was 3.63 m/s for the ***elastography maximum value (Emax)*** (Sensitivity 90%, Specificity 77.6%) and 3.44 m/s for ***Emean*** (Sensitivity 90%, Specificity 76.4%) (17). When analyzing ***pSWE*** optimal C/O for best performance at 2.87 m/s have been reported with a sensitivity of 75% and specificity of 95% (18).

The World Federation for Ultrasound in Medicine and Biology (WFUMB) published in 2017 a “white paper” with recommendations to try to standardize the values ​​of the different ***TE***. Its most significant results were for ***strain ratio (SR)*** with a ***C/O*** of 3.79, with sensitivity of 97.8% and specificity of 85.7%; they also report ***SR*** values ​​of 1.5 and up to 5 in their investigations (19). In the same consensus, the lack of homogeneity in terms of the values ​​and ***C/O*** for ***2D-SWE*** and ***pSWE*** was noted (19), these technologies will be addressed later in this study.

Until now, it has not been possible to establish a consensus with the guidance of relevant institutions such as RSNA, European Radiology, Asiatic Radiology or endocrinological societies such as ATA, ETA (European Thyroid Association), bringing together the top researchers in ***TE*** in order to standardize the values ​​to be applied in all ***TE***; an example of such consensus is the one published by the RSNA in relation to Liver Elastography, where regulations, conditions, cut-off levels and values ​​are presented and standardized measurement units between the different brands of equipment of ultrasound are established (20).

**Figure 1:**
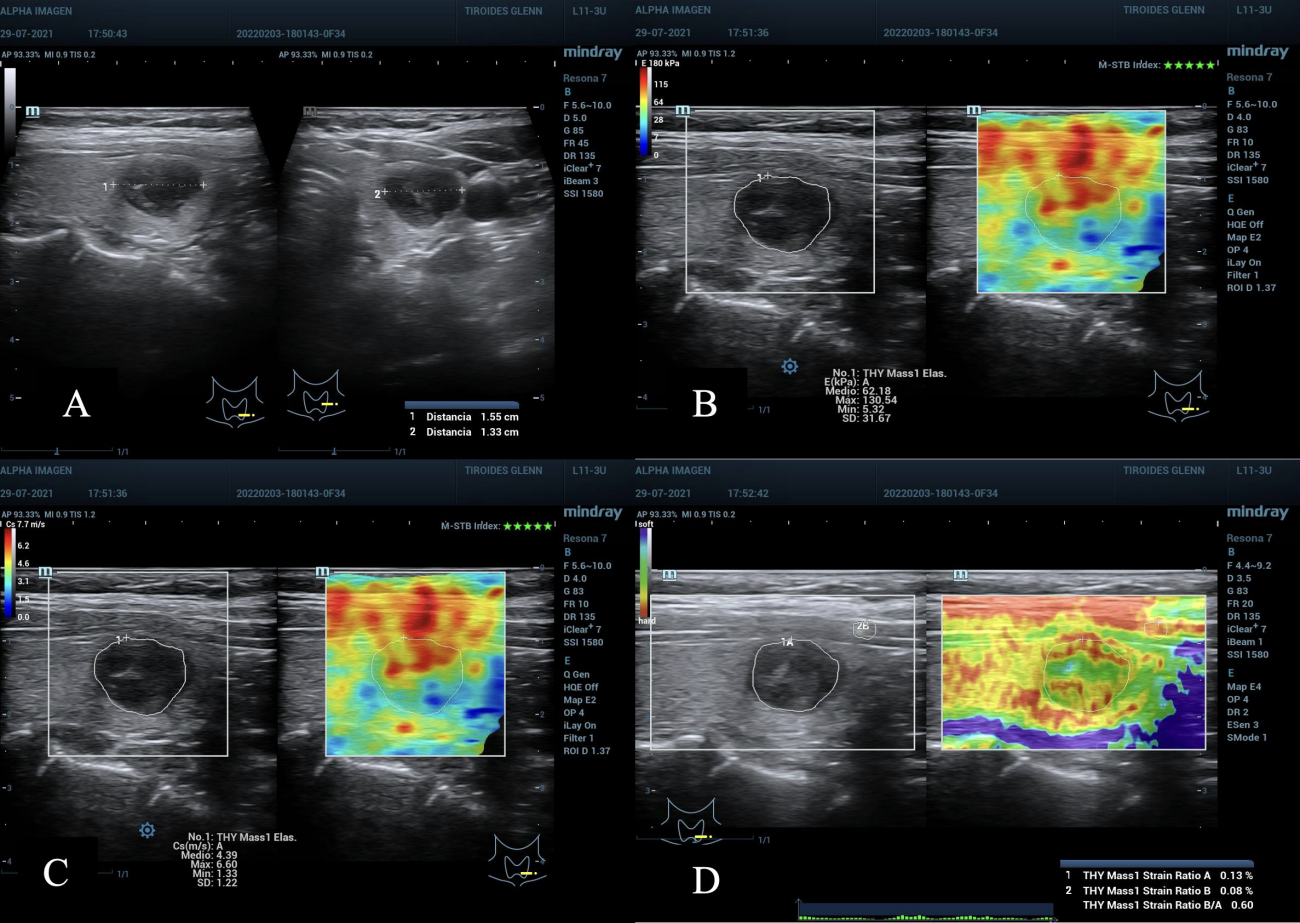
**(A)** Nodule in the lower left third of the thyroid gland. ACR TI-RADS 4, AS high suspicion, diameter greater than 1.55 cm. **(B, C)** TE 2D-SWE Emean and Emax above the C/O both in kPa and m/s. **(D)** SE A value of 0.13% suspicious for malignancy, and SR nodule/muscle of 0.6, lower than expected, not useful. Cytopathology: Bethesda VI. Post-surgical result: Papillary Thyroid Carcinoma.

**Figure 2:**
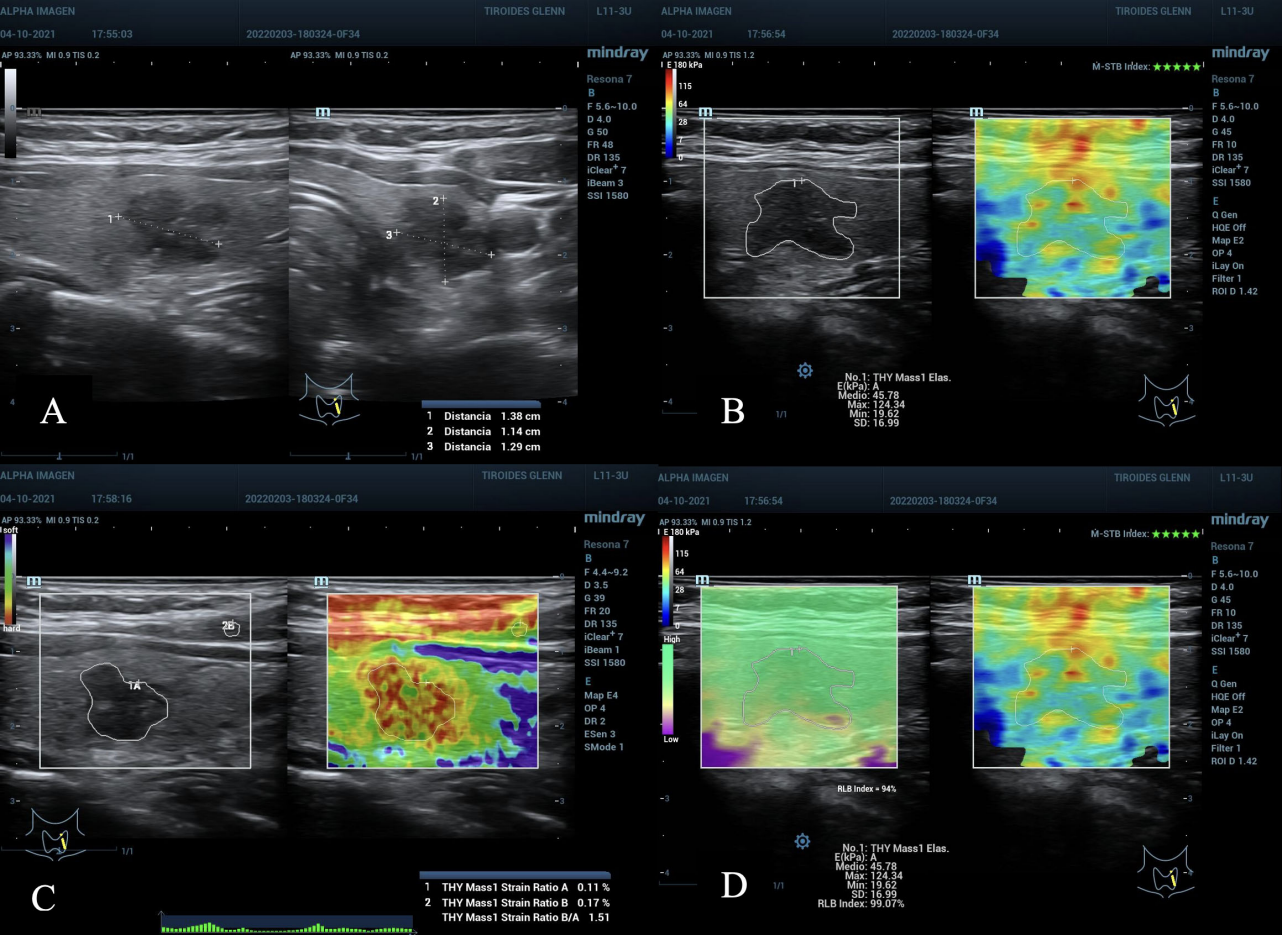
**(A)** Nodule in the left middle third of the thyroid gland, 1.38 cm in its largest diameter, ACR TI-RADS 4, AS high suspicion. **(B–D)**, TE 2D-SWE Emean, Emax above the C/O, A value 0.11% suspicious for malignancy, SR nodule/muscle slightly elevated. **(D)** quality maps, homogeneous green hue (optimum), M-STB Index with 5 stars and RLB index with 94%, values ​​considered optimal. Bethesda VI result, post-surgical histopathology: Papillary Thyroid Carcinoma.

**Figure 3:**
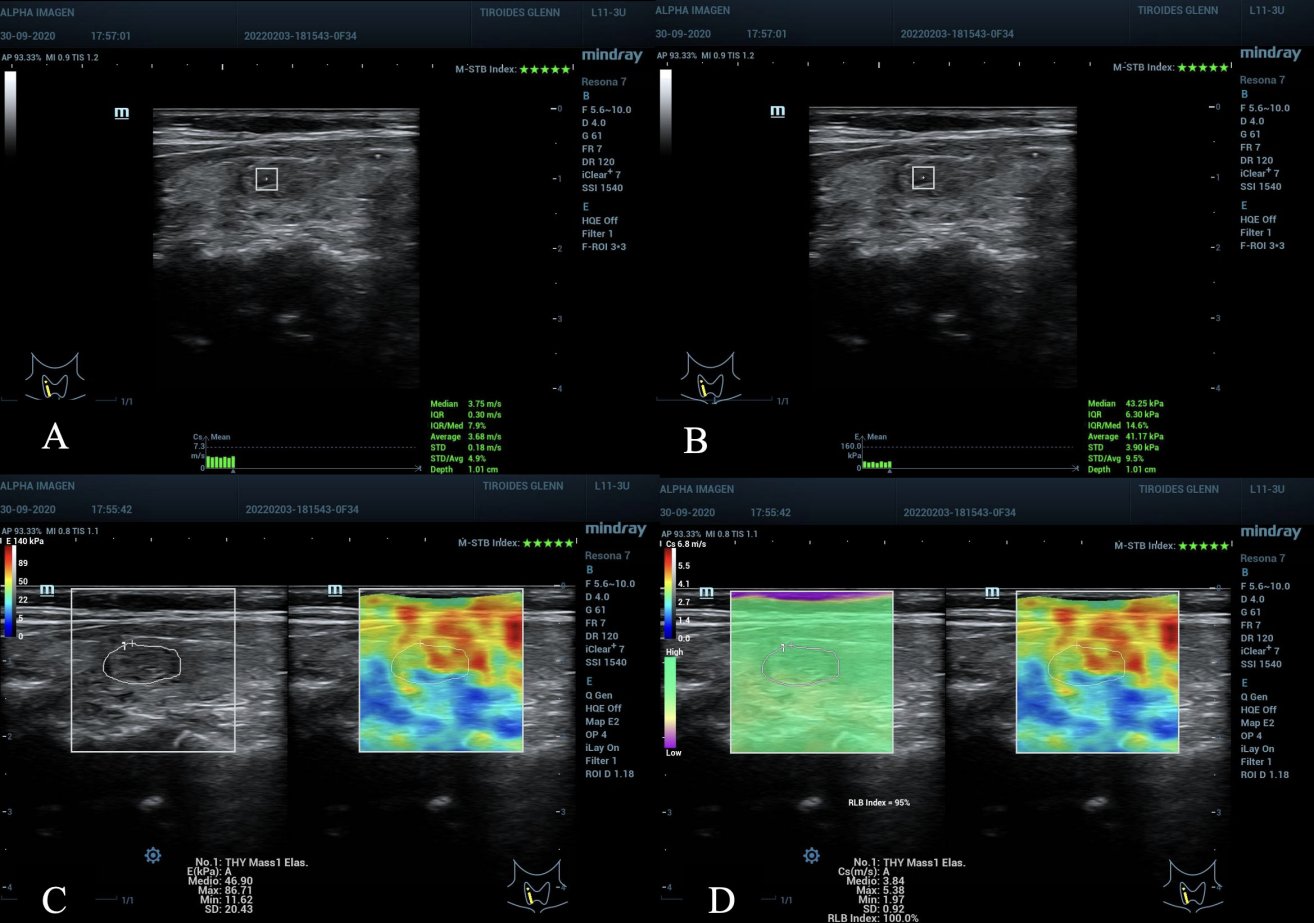
Nodule in the right middle third of the thyroid gland, ACR TI-RADS 3, AS low suspicion. **(A, B)** pSWE with values ​​below the C/O, observe the optimal MIQR values ​​for both kPa and m/s, 3x3 mm ROI box, 1.0 cm depth. **(C, D)** Emean in kPa, maximum scale 140 kPa, the C/O is slightly elevated, but not the values: E max kPa, E max m/s and E mean m/s that are below the C/O. **(D)** the maximum scale in m/s of 6.8 has been used, the quality maps M-STB Index with 5 stars and RLB index with 95% with optimal values ​​for obtaining the samples. Cytopathological result: Bethesda II, benign.

**Figure 4:**
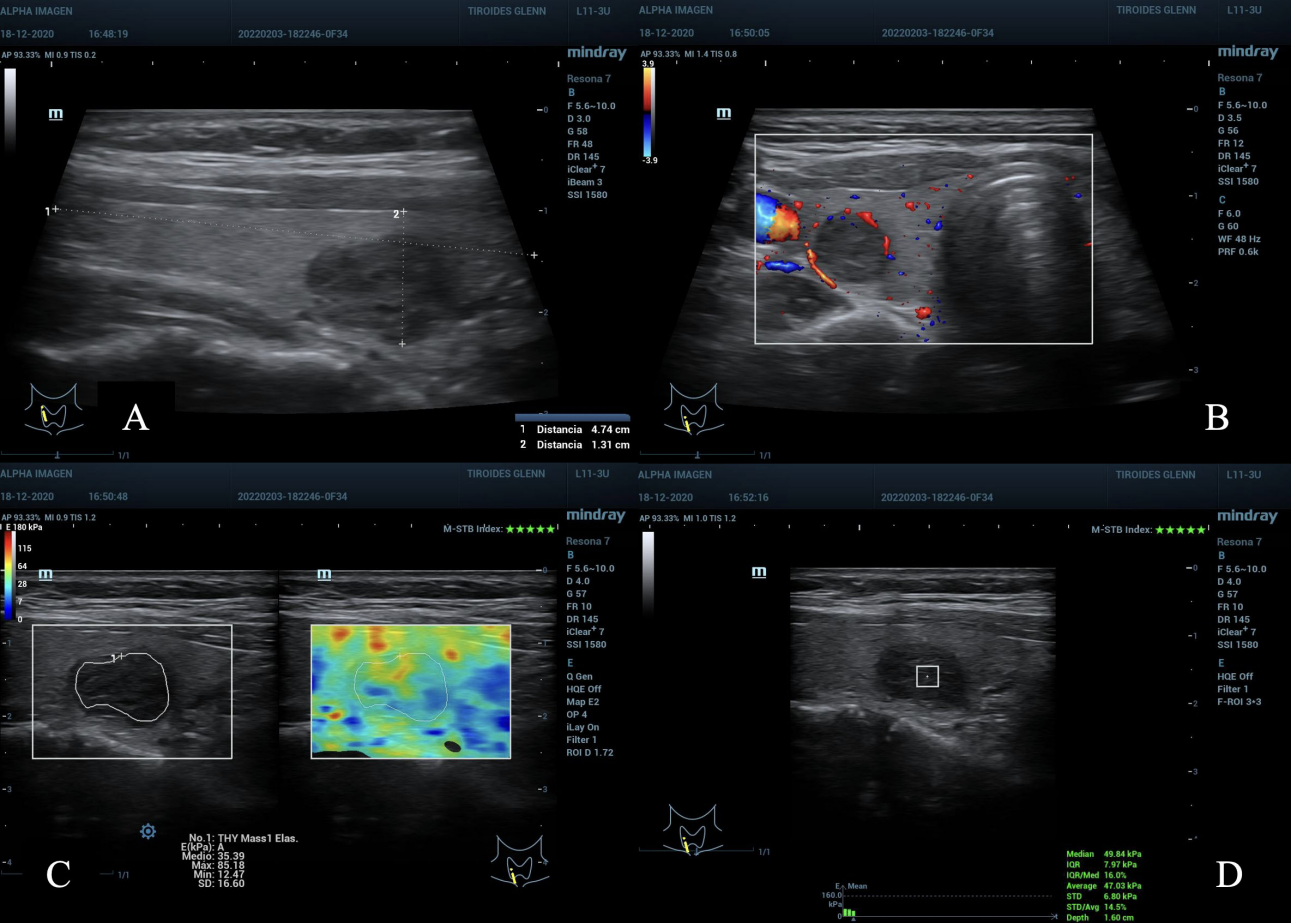
**(A)** nodule in the lower right third of the thyroid gland, maximum diameter 2.0 cm, ACR TI-RADS 4. **(B)** peripheral Doppler vascularization, AS moderate suspicion. **(C)** maximum scale of 180kPa, TE 2D-SWE Emean and Emax with kPa below C/O. **(D)** pSWE kPa below C/O, MIQR 16%, ROI box 3 x 3 mm, depth 1.6 cm. Result: cytopathological Bethesda: II, benign.

**Figure 5:**
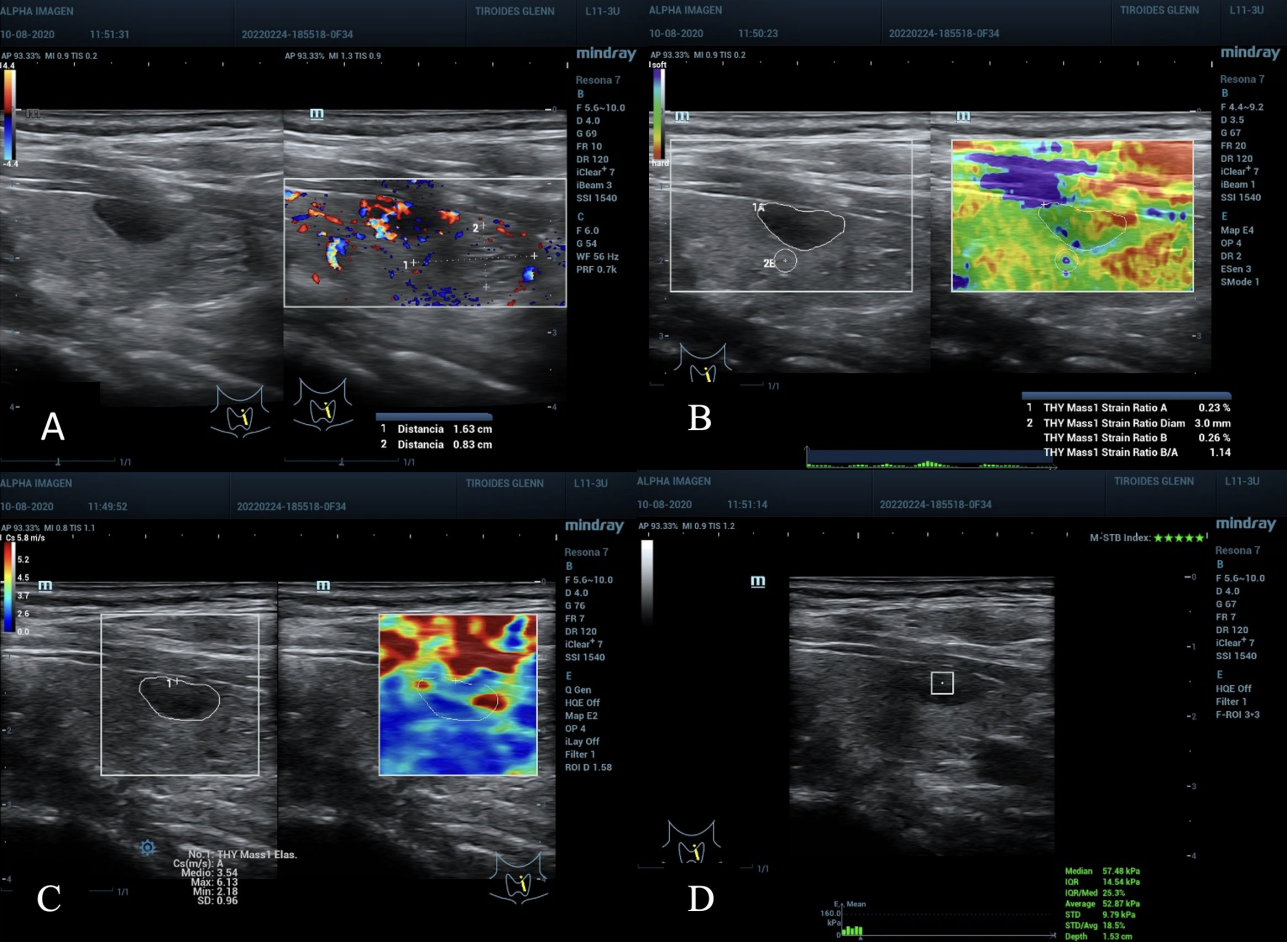
**(A)**, nodule in the left middle third of the thyroid gland, diameter greater than 1.6 cm, peripheral vascularization, ACR TI-RADS 4, AS moderate suspicion. **(B)** SE with ROI B of 3 mm, SR Nodule/Tissue of 1.14 under the C/O and Value A of 0.23% not suspicious. **(C)** 2D-SWE, full scale 5.8 m/s, Emean 3.5 and Emax 6.1 cm/s below C/O. **(D)** pSWE slightly above C/O, 52.8 kPa average, 57.4 kPa median, unreliable values ​​by the MIQR of 25% above the recommended value (15% for kPa), depth also at the maximum limit 1.53 cm. Cytopathological result: Bethesda II, benign.

**Figure 6:**
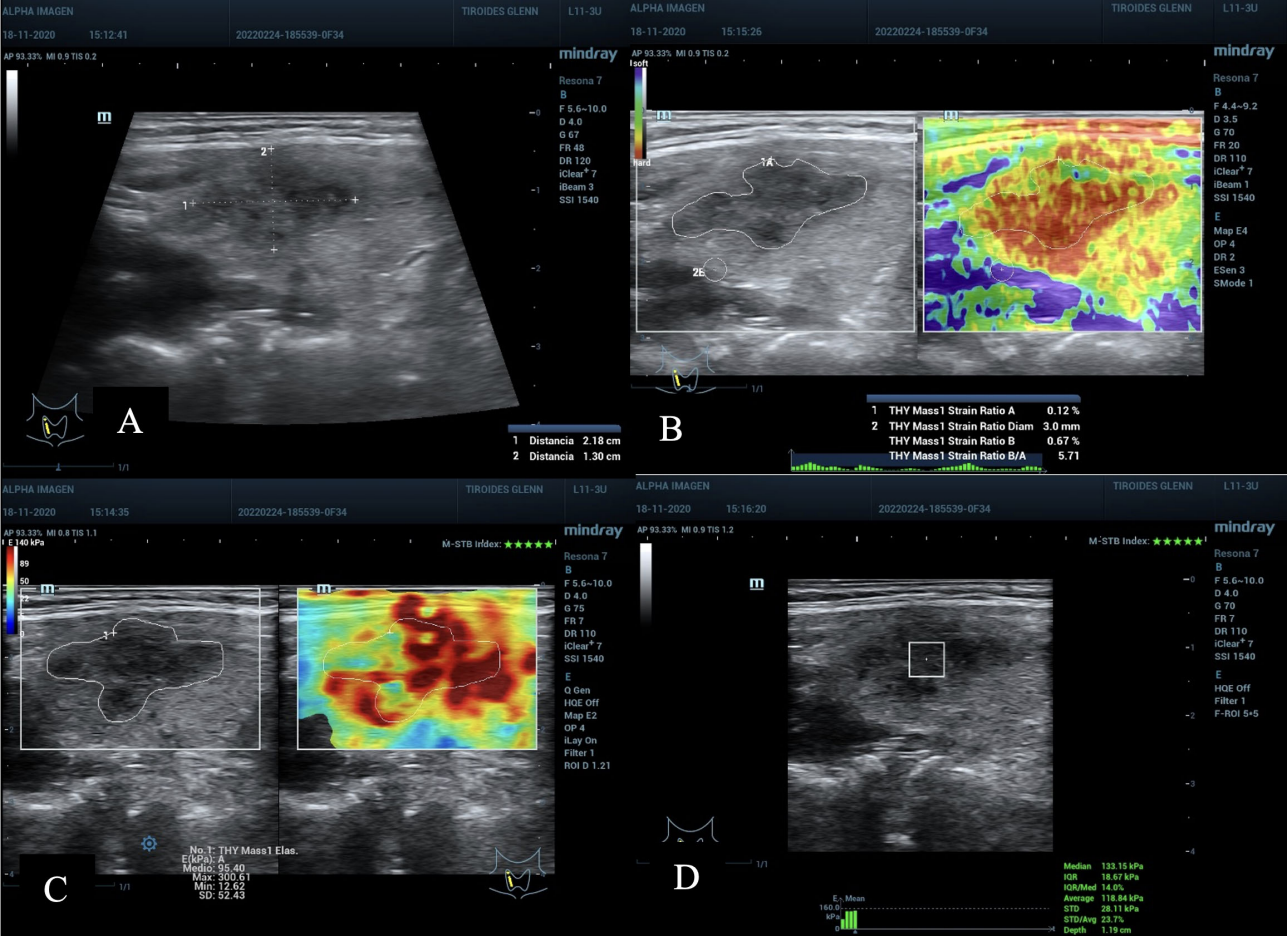
**(A)** nodule on the right upper third and middle of the thyroid gland, diameter 2.1 cm; ACR TI-RADS 5 and AS high suspicion. **(B)** SE with SR Nodule/Tissue of 5.7 above the C/O and value A of 0.12%, suspicious of malignancy. **(C)** 2D-SWE Emean 95.4 and Emax 300 Kpa, above C/O. **(D)** pSWE with 118 kPa (average) and 113 kPa (median), above the C/O, with a good MIQR of 14% and a good depth of 1.19 cm. Cytopathological result: Bethesda V, post-surgical result Papillary Thyroid Carcinoma.

Here, we present our results using three types of ***TE*** using the Mindray Resona 7^®^ model, an equipment in “Ultra-Wide Beam Tracking Imaging” technology that provides real-time processing of all signals in a target area from 0.2mm to 40mm (21); two of these TEs are quantitative and use SWE technology: real-time SWE and focused pSWE (21), and the third elastography, uses deformation SE technology (22) (**Figures 1**–**6**). In addition, we present important results with ***pSWE*** on the values ​​of the mean interquartile ratio (MIQR) for TN, for both kPa and m/s; we also discuss the recommended depth ranges for sample acquisition in centimeters (cm), the size of the boxes recommended for the Region of Interest (ROI), and the quality control values for SWE ​​known as “Reliability Index & Map” (RLB-Index & RLB-Map).

## Materials and methods

At the Institute of Radiology and Interventionism, Alpha Imagen, Quito, Ecuador, from January 2020 to December 2021, 196 TNs were analyzed, all of them had fine needle aspiration biopsy (FNAB) by different specialists; all TNs were classified by ultrasound using two types of malignancy predictors: ACR TI-RADS and Alpha Score (AS), a previously reported US prediction score validated in Latin-American countries (23, 24). Of those, 170 TNs with benign (Bethesda II) or malignant results (Bethesda V and VI, verified with post-surgical histopathological results) were selected. TNs with Bethesda I results were eliminated due to insufficient number of samples; similarly, Bethesda III and IV nodules without a definitive histopathological result were also eliminated. All patient data was anonymized for analysis and the study received approval from the Institutional Review Board (AI-ET-2021).

A Mindray^®^ brand ultrasound equipment, model RESONA 7^®^, was used, equipped with a multifrequency linear transducer model L 11-3U with a frequency range of 3 to 11 MHz and a central frequency of 7 MHz; equipped with thyroid elastography software type ***SE***, ***2D-SWE*** and ***pSWE***. The same soft tissue protocol was used for optimal evaluation of the thyroid gland. In the equipment, the scales that can be seen on the left side of each image represent the interval between the minimum and maximum values ​​that can be used in that measurement (in our equipment these values are 10-400 kPa and 1.8-11.5 m/s). The color scale available for the ***SWE*** allows establishing an interval with the possible values ​​to be found during the exams and can be manually modified. It is worth remembering that when the colorimetric scale is used as an orientation to see the areas of greater hardness of the TN, the scale can be modified until the desired visual balance is found, this does not affect the TE measurement values, they only help to visually differentiate areas with different elasticity. We measured the complete integrity of the TN, its entire circumference without considering the areas of lesser or greater hardness, that would be represented by the machine with different shades of color.

The following measurement modules were used in this equipment:

**Young’s modulus,** based on the equation E = σ/ϵ where E is the modulus of elasticity expressed in kPa, σ is the stress, and ϵ the strain (25).

**Shear Modulus,** based on the equation G = pcs2 where G is the shear modulus expressed in kPa, p is the tissue density expressed in kg/m3 and Cs is the shear wave velocity expressed in m/s (25).

**Young’s modulus and Shear modulus relationship** is equal to , where the Young’s modulus of elasticity E is three times the shear wave modulus G (25).

The control and reliability tools used in this study were:

**Elasticity bar:** There is an elasticity bar which indicates, with a green color, if the obtaining of the sample is acceptable.

**Motion Stability Index/M-STB Index: *TE*** can be affected by breathing or movement of the transducer, so a motion stability analysis tool is available at the time the measurements are made. It is formed by a scale of 5 stars, from 1 to 3 stars there is movement and the ***TE*** measurement should not be carried out since the values ​​obtained will be erroneous; in contrast, from 4 to 5 stars it indicates that the measurement must be carried out since the external movement is null.

**Reliability Index & Map/RLB-Index & RLB-Map:** The indicator and reliability map indicate the homogeneity of the sample box; in this case the value must be greater than 90% (92% in our study) and the green map indicates that we have a sample without artifacts.

In our study, all benign and malignant TNs were analyzed with the three types of ***TE***, classified with ACR TI-RADS and AS, and correlated with Bethesda cytopathological results and histopathological results in malignant cases. These comparisons will result in high reliability of the results.

The interpretation was performed by two radiologists with more than 5 years of experience in thyroid ultrasound diagnosis and training to perform FNAB, use of ACR TI-RADS and AS. Data collection, and imaging acquisition was performed by a medical technologist in radiology trained for the processing of both scores, as well as for the processing of images, and data analysis.

FNABs were always obtained hands-free with an MD TECH ^®^ brand vacuum cytoaspirator and a 20 ml syringe with a 23 g 1 ¼ inch needle. The samples were prepared in dry slides for Giemsa studies and others were fixed in absolute ethanol for Papanicolaou, a part of the sample was sent in base cytology carrier liquid for its cytopathological process and/or as a cell block (histopathological); the malignant cases (n= 46), all managed surgically, had a confirmation of the malignant lineage through histopathological assessment.

The complete measurement of the entire contour of the nodule was performed with ***TE 2D-SWE***, following its internal border, without exceeding its external contour, including all the content, whether solid or mixed; the tracing was made manually by the operator, to determine the measurements of Young’s modulus with values ​​of ***Emax***, ***Emean*** and ***elastography minimum value (Emin)*,** in kPa and m/s units and the values ​​of the colorimetric scale used in kPa were also selected. Measurements with ***pSWE*** were made inside the nodule with a modifiable rectangular or square ROI, whose limits should not exceed any of its edges. All of the different textures of the TN were included inside the ROI, either solid or mixed; the largest possible ROI that could fit inside the nodule was used. The automatic values ​​of the multiple pSWE pulse sequences (up to 8) provided multiple measurements and the best average values ​​(Average) and the value of the median, both in kPa and in m/s, provided by the machine itself were used (**Figures 3** **–** **6**). Additionally, depth values ​​in cm, the size of the ROI in mm x mm, the value of the MIQR for kPa and for m/s, and the values of the kPa and m/s scales when using ***pSWE***, were also collected.

When using the ***SE*** technology, with the transducer supported by its own weight, without significant compression of it, the following measurements were made: using the complete contour of the nodule, traced manually and following its internal edges, the percentage of deformation of the nodule (***value A***) was obtained and compared with a circular ROI of fixed size (3 mm) located in the healthy thyroid tissue (***value B)*** and oriented by the color map to choose an area with the least hardness (in this equipment it is represented with a light blue color). For a second comparison, ***value A*** was obtained in a similar fashion, but an area in the ipsilateral sternocleidomastoid muscle (SCM) was chosen for ***value B***, which was included within the ***SE*** measurement box (a ROI of up to 3 mm was manually traced). The ***B/A ratio*** in both cases was determined automatically by the equipment and used to determine the ***SR Nodule/Tissue (SRN/T)*** and ***SR Nodule/Muscle (SRN/M)***, respectively.

All measurements were saved in the equipment and in a digital image storage system (PACS) for future reviews and comparisons, all studies were again randomly reviewed by the two expert radiologist and by the medical technologist. This analysis resulted in the creation of a TN database that included all of those cases with an adequately acquired measurement and eliminating those with an incomplete or inadequately acquired measurement.

**Eligibility criteria:** Patients sent to perform a FNAB by specialists in thyroid pathology, and that have not received radioactive iodine therapy, nor have a history of previous surgery or FNAB in the last 3 months. In the case of patients with multinodular pathology, only the TN with the highest score obtained in AS and ACR TI-RADS was selected. Patients who had received radioactive iodine treatment, previous surgery, or any other intranodular treatment such as sclerosis or radiofrequency were excluded, as well as those who underwent FNAB in the last 3 months.

**Statistical Methodology:** A prospective simple random sampling was performed based on the total number of ultrasounds performed in “Alpha Imagen” in the previous 18 months and considering a reported national thyroid cancer prevalence of 15%; this resulted in a sample size of 178 subjects (which includes a 15% surplus in case of missing data or loss of participants), which should be enough to estimate the variable of interest with 95% confidence and precision of +/- 5 percent units. The evaluation of the diagnostic capacity and the efficiency of the software used was carried out, including a total of 170 TNs from 170 patients studied in “Alpha Imagen”, whose participation was voluntary and affirmed by the signature of an informed consent. Data was collected in units of m/s or kPa, grouped into ***SWE***, ***pSWE*** and ***SE***, which determined the following measurements: ***SWE*** kPa ***Emax***, ***SWE*** kPa ***Emean***, ***SWE*** kPa ***Emin***, ***SWE*** m/s ***Emax***, ***SWE*** m/s ***Emean***, ***SWE*** m/s ***Emin***, median ***pSWE*** m/s, average ***pSWE*** m/s, median ***pSWE*** kPa and average ***pSWE*** kPa, ***Value A***, ***SRN/T*** and ***SRN/M***. The data series were initially used to calculate the graphs of the ROC curves, evaluating the distances with the diagonal and the area under the curve (AUC). To determine the best level of the indicators, various ***C/O*** were made using the technique of successive approximations. The calculation of the diagnostic tests used the “MSD Calculator professional version” (26). For the quantitative, nominal and continuous variables, absolute and relative frequencies were used. The assumption of normality for continuous data was validated using the Shapiro-Wilk test. The data was refined by computerized identification of atypical cases; the intergroup differences of men and women were analyzed through the comparison test of means (T test) and (ANOVA). All the analyzes were carried out with the SPSS statistical package, version 25. The required complementary evaluation was examined through the ROI, MIQR and RLBINDEX indicators, for which the point statistics were generated using measurements of central tendency: Means, Medians, Modes, Standard Deviations, Skewness, Mean Deviation Error and Ranges. The statistical results were analyzed by the entire group of authors in different meetings to establish the clinical-radiological and statistical correlations and to be able to obtain the different ***C/O*** for benign and malignant TNs.

The entire process was supervised and verified by an experienced statistician from our imaging institute and by a biomedical engineer with experience in the brand’s applications and technology.

## Results

The total number of thyroid nodules studied during the aforementioned time period was 195, from which 170 TN were selected; Bethesda I (n=3), III (n=15) and IV (n=7) were excluded in order to work with only benign lesions diagnosed by cytopathology or malignant nodules confirmed by histopathology. The cytopathological results of the included TN were Bethesda II, V and VI, of which 46 patients were confirmed as malignant after post-surgical histopathological analysis. The included participants were in their majority women, representing 88.8% (n=151) of the sample with a mean age of 51.8 years and males represented 11.2% (n=19) with a mean age of 56.9 years; the mean age for the entire cohort was 52.2 years.

In our study, scales with maximum values ​​from 75 kPa to 400 kPa were used, finding that the most frequently used were the maximum scale of 180 kPa (in 48.5% of cases) and that of 140 kPa (in 34.1% of cases). Considering that the average values ​​found in our study range from 13 kPa (***Emin***) to 115 kPa (***Emax***), these scales perfectly cover the biometric requirements of the nodules. When the scale is changed to units in m/s, the most used maximum scales were 6.5 m/s (in 42.4% if cases) and 7.7 m/s (in 41.8% of cases); we recommended to use the latter, which would cover all the average values ​​found between 2.0 m/s (***Emin***) and 6.5 m/s. (Emax). Regarding the kPa scale, it is shown that the majority of benign TN (Bethesda II) are located in maximum scales of up to 140 kPa, while Bethesda V are located in the range of 140 to 160 kPa, and VI are generally located in scales greater than 160 kPa. Comparing the Bethesda scale with the speed in m/s, it is noted that nodules with a speed of up to 6.4 m/s would qualify as benign (Bethesda II), between 6.5 and 7 m/s would be Bethesda V, and 7 m/s or higher, Bethesda VI. No significant differences were found between the results obtained for the TE values ​​regardless of the scale used (**Figures 1** **–** **6**).

**Table 1** shows the ***C/O*** and the diagnostic tests of the different types of quantitative TE used in this study, six with ***SWE*** and four with ***pSWE***. When analyzing all of the elastographies together, the overall diagnostic performance was: sensitivity of 81.2%, specificity of 57.6%, PPV of 72.4%, and NPV of 70.4%.

**Table 1:** Diagnostic tests: sensitivity, specificity, PPV, NPV by Cut-Off Points according to the type of measurements for Shear Wave and Point Shear Wave.

Elastography Type	Point Statistics					
	Cut-Off Value		Sensitivity %	Specificity %	PPV %	NPV %
2D-SWE kPa Emax	115		79.57	64.74	73.27	72.46
2D-SWE kPa EMean	47.5		83.16	59.46	72.48	73.13
2D-SWE kPa and Emin	13.0		75.27	70.13	75.27	70.12
2D-SWE m/s E max	6.5		84.62	56.92	73.33	72.55
2D-SWE m/s E mean	4.0		84.31	55.88	74.14	70.37
2D-SWE m/s E min	2.0		64.94	79.5	75.76	69.66
pSWE kPa Median	52.6		83.84	57.75	73.45	71.93
pSWE kPa Mean	52.4		80.2	62.32	75.7	68.28
pSWE m/s Median	4.15		81.37	58.57	74.11	68.33
pSWE m/s Mean	4.1		75.47	64.38	75.47	64.38

Source: Alpha Elastography Image Database 2021 The results of the diagnostic tests obtained for two-dimension Shear Wave (2D-SWE) and Point Shear wave (pSWE) are detailed in units (kPa or m/s) and with its different values ​​in Young’s modulus, Emax, Emean, Emin for 2D-SWE and mean and median value for pSWE. NPV, Negative Predictive Value; PPV, Positive Predictive Value.

Our analysis of ***Strain Elastography (SE)*** resulted in a ***C/O*** for ***value A*** of 0.20% with a sensitivity of 84%, specificity of 57%, PPV of 72% and NPV of 73% (**Tables 2**, **3**). Additionally, when looking at ***Strain Ratio (SR)***, we propose a ***C/O*** of 2.69, yielding a sensitivity of 84%, specificity of 57%, PPV of 72%, and NPV of 73% **(** **Tables 2**, **3** **).** When analyzing these ratios, in order to compare the difference of using ***SRN/T and SR N/M***, we repeated the measurement process in all nodes; this time we chose ***value B*** in the tissue closest to the ***SCM*** muscle and used a circumferential manual ROI of 2 to 3 mm in a sector free of pathology. In those cases, ***value A*** was very close to what we obtained when assessing the ***SRN/T*** (0.20%), this time it was 0.19% (sensitivity 81%, specificity 61%, PPV 70% and NPV 74%). These showcases our degree of interobserver reproducibility in the circumferential measurement of the TN since it was performed randomly by all the experts in this study. The ***SRN/M ratio*** obtained was 1.15 (sensitivity 82%, specificity 65%, PPV 82% and NPV 64%) (**Table 3**). We do not consider this ***SR*** the most appropriate, since it has a standard error of the mean of 0.10 and a SD of 1.31 (**Table 2**).

**Table 2:** Strain Elastography, Value A, Nodule/Muscle Strain Ratio and Nodule/Tissue Strain Ratio.

Elastography of Thyroid Nodules	Value A Relationship Nodule/ Muscle	Value B Relationship Nodule/ Muscle	Values B/A ratio SRN/M	Value A Relationship Nodule/ Tissue	Value Relationship Nodule/ Tissue	Values B/A ratio SRN/T
Valid	169	168	167	167	167	167
Lost	1	2	3	3	3	3
Mean	**0.19**	**0.18**	**1.15**	**0.20**	**0.50**	**2.69**
Standard error of the mean	0.006	0.011	0.10	0.0069	0.01679	0.08
Median	0.17	0.15	0.85	0.18	0.46	2.32
Mode	0.15	0.14	0.41	0.15	0.41	1.71
Standard Deviation	0.08	0.15	1.31	0.08	0.21	1.15
Minimum	0.07	0.03	0.17	0.09	0.2	1.04
Maximum	0.51	1	11.8	0.59	1.89	7.57

Note: The mean of the results indicates the C/O found for the main values ​​obtained, the TN Value A, the Strain Ratio SRN/M (nodule/muscle) and the Strain Ratio SRN/T (nodule/tissue). Note how value A ​​have fairly close results and correspond to measurements made by different observers. The value B does not have a radiological clinical meaning in this study since it’s a tissue sample (in the thyroid or in the ECM) to obtain the B/A ratio of the Strain Ratio.

The value is the mean of the Strain Value A divided by value B of all the thyroid nodules; it has no units.

**Table 3:** Diagnostic tests obtained with Strain Elastography analysis according to Strain measurements in tissue, muscle and the ratios with tissue and muscle.

STRAIN ELASTOGRAPHY	SENSITIVITY	SPECIFICITY	PPV	NPV
VALUE A (SR nodule/muscle) 0.19	0.81	0.61	0.7	0.74
VALUE B (SR nodule/muscle) 0.18	0.79	0.59	0.765	0.755
VALUE B/A (SR nodule/muscle) 1.15	0.82	0.65	0.824	0.64
VALUE A (SR Nodule/Tissue) 0.20	0.84	0.57	0.724	0.736
VALUE B (SR Nodule/Tissue) 0.50	0.85	0.65	0.786	0.736
VALUE B/A (SR Nodule/Tissue) 2.69	0.84	0.57	0.7238	0.735

Source: Alpha Database Image Elastography 2021

Note: Consider of greater importance the tests obtained in the results of the value A that correspond to the TN,

as well as to ratios, the value B is just the comparative tissue value. It is worth noting that the TN elasticity strain value is quite similar to the SCM strain value, which explains its close SR at 1.0, but not so with the strain value in the thyroid tissue, which reaches higher values ​​and therefore its SR increases, which is why SRN/T is considered the best option. NPV: Negative Predictive Value, PPV: Positive Predictive Value.

When assessing quantitative TE, we managed to obtain good statistical results with both ***SWE*** as well as with ***pSWE***, using values in kPa, m/s, ***Emax***, ***Emean***, mean and median; the differences were not significant and the values were between 64.3%-84.6% for sensitivity, between 55.7-77.6% for specificity, between 70.1%-73.5% for the PPV and between 64.4%-73.2% for the NPV **(** **Table 1** **)**. One of the most commonly used measurements, the ***SWE Emean***, had a C/O of 47.5 kPa, with a sensitivity of 82.9%, specificity of 56.8%, PPV of 70.1%, and NPV of 73.2% **(** **Table 1** **)**. When analyzing values on m/s we found an ***Emean*** value of 4.0 m/s using ***SWE*** (sensitivity 84%, specificity 55.8%, PPV 74.1% and NPV 70%), an ***SWE Emax*** of 6.5 m/s with a sensitivity of 84.6%, specificity of 56.9%, PPV of 73.3%, and NPV of 72.5%; and average ***pSWE*** of 4.15 m/s (sensitivity 81.3%, specificity 58.7%, PPV 74.1%, and NPV 68.3%) **(** **Table 1** **).**

In addition to the diagnostic analysis, we also managed to stablish some technical parameters that can serve as a guide for the correct use of the ***TE*** and for comparison with future research. Thus, we recommend using intervals between 140 and 180 kPa as the maximum limit at the top of the colorimetric scale when using ***SWE*** and 7.7 m/s when using the scale in m/s, this results in optimum color and velocity maps considering an ***Emax*** cut-off value between 115 kPa and 6.5 m/s. Using a larger scale would not be recommended, it is unnecessary, and it also generates color patterns in the 2D map that are not very useful; additionally, speed scalation intervals become very wide. When considering the size of the ROI box in ***pSWE***, the ones we used the most were the 3x3 mm and 5x5 mm; however, this was highly dependent on the nodule size since we used a ROI that included the TN without exceeding its external borders, therefore the size of the ROI will depend fundamentally on the size of the nodule. The nodule sizes found in our study had an average diameter of 1.71 cm ( ± 0.871 cm; SD) ranging from 0.6 cm to 4.0 cm; the ROIs were between 1 x 1 mm and 20 x 20 mm with an average of 5x5mm. No significant differences were found between the results obtained from the ***TE*** values ​​regardless of the ROI size used (**Table 4**). Another aspect to consider is the value of depth in cm, in our case the average acquisition depth of the ***pSWE*** sample was 1.38 cm and the mean depth was between 1.28 and 1.53 cm; note that in the malignant TN the mean was 1.47 cm (**Table 4**). There were no significant differences between the depth of benign and malignant TN; even though a depth of acquisition of 1.2 to 1.5 cm is recommended, the use of other values does not result in significant differences when comparing benign and malignant TNs.

**Table 4:** Measurements of central tendency by region of interest (ROI) size and sample acquisition depth using pSWE.

ELASTO pSWE in cm		GLOBAL Benign and Malignant	ROI 3x3 and 5x5mm	ROI 3x3mm	ROI 5x5mm	Malignant TN
N	Valid	170	116	38	78	46
	Lost	0	0	0	0	0
**Mean(cm)**		**1.37**	**1.36**	**1.53**	**1.28**	**1.47**
Standard Error of the Mean		0.03	0.03	0.06	0.03	0.06
Median		1.32	1.34	1.45	1.29	1.40
Mode		1, 2	1.29	1.00	1.5	1.24
Standard Deviation		0.42	0.37	0.039	0.33	0.41
Minimum		0.38	0.55	0.79	0.55	0.88
Maximum		2.87	2.26	2.64	2,222	2.51

Source: Alpha Database Image Elastography 2021

Note: ROI: region of interest, TN: thyroid nodule. Cross analysis of the depth in cm of the acquisition of the samples with pSWE and the different sizes of the ROI boxes. The mean should be considered as the best test statistic in this table.

The bold values represent the mean depth of the acquisition in centimeters for all TN, for the 3x3 and 5x5 ROIs together, for the 3x3 and 5x5 ROIs individually, and for only the Malignant TN.

With regards to the RLB INDEX, despite reports indicating that its optimal preset value is ≥90%, we recommend that it should be ≥92%. In our study, there were no significant differences between benign and malignant TN when using this value, which informs us of its usefulness in standardizing the measurements performed in our equipment (**Table 5**).

**Table 5:** Central tendency measurements of the Reliability Index (RLB INDEX) by Total, Benign, and Malignant TN according to type of measurement.

Statistics	Total	Benign	Malignant
N	170	124	46
**Mean%**	**92.59**	**92.81**	**92.02**
Standard Error of the Mean	0.78	0.95	1.33
Median %	96	97	94
Mode %	100	100	100
Minimum %	51	51	63
Maximum %	100	100	100

Source: Alpha Database Image Elastography 2022

Note: It has been found that the RLB INDEX quality control must have a minimum mean value of 92%, note that for benign or malignant TN the values ​​are similar for the mode, but not for the standard error where in malignant it was higher (1, 3) for which the largest possible value of RLB INDEX is needed. For practical purposes, a value lower than 92 is not recommended, thus ensuring an optimal measurement.

The bold values represent the mean result of the Reliability Index which is expressed as a %. So, these are the mean values for the total, benign, and malignant TNs.

Our analysis showed that the relationship of the MIQR between kPa and m/s is almost doubled, specifically 1.9 for all 170 TNs, 1.86 for the benign nodules and 2.1 for the malignant ones; therefore, for all TNs (benign and malignant) the recommended value would be 8.1% for m/s and 15.7% for kPa, although for malignant TN these values may be higher with means of 9% for m/s and 19.2% for kPa; we would recommend to use the standard values of 8% for m/s and 15% for kPa (**Table 6**).

**Table 6:** Mean Interquartile Index (MIQR) by type of central tendency measurement, according to scales (kpa/m/s) for benign and malignant TN.

MIQR in m/s	Mean	Mean Standard Error	Median
TOTAL (170)	**8.16**	0.48	6.7
BENIGN (124)	7.75	0.4	6.65
MALIGNANT (46)	9.23	1.2	7.45
MIQR in kPa			
TOTAL (170)	**15.7**	0.88	13.15
BENIGN (124)	14.89	0.82	13.2
MALIGNANT (46)	18.2	2.39	12.2

Source: Alpha Database Image Elastography 2021

Note: We present the recommended values ​​of MIQR both in m/s and in kPa, so far values ​​less than 30% using kPa and less than 15% using m/s are standardized for Liver TE. Roughly, we found that for TN the values ​​are about half, 15.7% for kPa and 8.1% for m/s.

The bold area represents a title within the table meaning that all the values below represent the mean (first column), the mean standard error (second column), and the median (third column) of the mean interquartile range (MIQR) but in kPa instead of in m/s as in the values above.

Lastly, the statistical analysis between Bethesda, ACR TI-RADS and AS showed correlation between the three classifications, even though Bethesda is considered the gold standard for pathological classification. **Table 7** shows the values of sensitivity, specificity, PPV and NPV between the three.

**Table 7:** Diagnostic tests of Two-Demensional Shear Wave Elastography (2D-SWE) and Point Shear Wave (pSWE) vs. Bethesda, ACR TI-RADS and Alpha Score.

SCALES	Bethesda				ALPHA SCORE				ACR TI-RADS			
Elastography Type	Sen	Spe	PPV	NPV	Sen	Spe	PPV	NPV	Sen	Spe	PPV	NPV
**2D-SWE** **kPa Emax.**	79.6	64.9	73.3	72.4	74.1	68.9	82.1	57.9	75.8	64.0	79.2	59.4
**2D-SWE** **kPa Average**	83.1	59.4	72.4	73.1	79.9	60.0	76.1	65.0	79.8	67.2	83.4	61.6
**2D-SWE** **kPa Emin.**	75.2	70.1	75.2	70.1	76.5	67.8	70.9	74.0	76.5	75.0	80.6	70.1
**2D-SWE** **m/s Emax.**	84.6	56.9	73.3	72.5	79.4	59.2	80.1	58.1	80.5	66.7	85.6	58.1
**2D-SWE** **m/s Emean**	84.3	55.8	74.1	70.3	81.0	55.7	76.7	62.9	82.0	52.4	76.1	61.4
**2D-SWE** **m/s Emin.**	64.9	79.5	75.7	69.6	60.2	77.4	71.2	73.0	67.9	83.1	80.3	71.9
**pSWE** **kPa Median**	83.6	57.7	73.4	76.9	81.1	57.8	76.1	64.9	81.5	64.3	82.3	63.1
**pSWE** **kPa Average**	80.2	58.5	74.1	68.3	804	58.7	76.7	63.8	78.1	65.4	83.1	58.0
**pSWE** **m/s Median**	75.4	64.4	75.4	64.3	77.7	62.9	78.5	61.9	77.4	71.7	85.8	59.3
**pSWE** **m/s Average**	75.5	64.4	75.5	64.4	77.8	62.9	78.5	61.9	77.8	71.7	85.9	59.4
**SR Nodule/Muscle**	72.4	77.8	68.8	80.7	71.4	72.8	68.8	75.3	76.0	75.5	71.3	79.8
**Value A %**	74.4	70.9	76.1	72.3	73.6	69.6	76.1	77.5	77.5	75.0	69.2	81.8
**SR Nodule/Tissue**	78.2	63.1	79	62.0	80.2	62.1	76.4	67.2	82.3	66.1	79.2	70.4

Source: Alpha Database Image Elastography 2022.

Sen, sensitivity; Spe, specificity; PPV, positive predictive value; NPV, negative predictive value. Two-dimensional shear-wave elastography (2D-SWE); Point Shear wave elastography (pSWE), A, elastic deformation of the TN.

## Discussion

Here we present an analysis of the diagnostic capacity of ***TE*** to differentiate malignant and benign TNs and propose ***C/O*** to enable such distinction; the main results can be found on **Table 1** and **Table 7** showcases the comparison of the diagnostic performance of ***TE*** against commonly used prediction scales and our own AS scale, that has been previously reported (23, 24). One of the first reports on the use of ***TE*** reported an exceptionally high AUC of 0.94 (16), but later studies did not managed to reproduce it; we believe that one reason lies with the fact that there is a lot of diversity in the different measurements and parameters that can be obtained in ***TE*** such as the definition of ROI, the type of ***SR***, the ***C/O*** and its values ​​with ***Emax, Emean***, the elasticity scale settings, and the scan planes used. This leads to a lack of consensus regarding the appropriate values ​​and parameters to be applied, regardless of the brand of sonography equipment being used.

When looking at ***Strain Elastography (SE)***, we have considered that the analysis of the entire content of the nodule can provide more useful information by allowing other observers and researchers to obtain similar results by establishing the complete measurement of the TN circumference as a fundamental parameter. According to our experience, measuring only the solid area has many inter-observer errors, it is not always uniform, it discards thick areas, it requires more expertise on the technique, and is time consuming leading to less reliability of the measurements.

Published studies report an AUC for ***SE*** and ***elasticity contrast index (ECI)*** that ranges between 0.61 to 0.94 with a specificity and sensitivity that range between 48% to 97% and 42% to 95%, respectively (14). Therefore, although several studies provide ***C/O*** levels of the ***ECI*** that can be easy to use on a group basis, the diagnostic value in the individual patient is suboptimal, which is explained by the large overlap of results between benign and malignant TNs (14). Regarding the value provided by our analysis of ***SE***, we found significant findings with a ***C/O*** of ***value A*** at 0.20% (**Tables 2**, **3**). A previous study using the same make and model as ours reported a value of 0.215% with sensitivity, specificity, positive likelihood ratio (LR+) and negative likelihood ratio (LR−) of 71%, 73%, 2.58 and 0.40, respectively; quite close to ours (16).

When ***SR*** is used, ***C/O*** values ​​>2.32 have been reported with a sensitivity of 95.2% and specificity of 86.5% (18), using the relationship of the ***SR*** between the inner edges of the lesion as ***value A*** and taking the ***value B*** to be an area of ​​healthy thyroid tissue, that is, similar to our ***SRN/T***. In another study, a longitudinal and axial measurement was performed in the TN, as well as the ***value A*** inside the nodule versus muscle and normal thyroid tissue, finding better values ​​in the axial measurement and when ***value B*** was used in the thyroid tissue, the optimal ***C/O*** was 0.17% for ***value A*** and 2.66 for ***SR*** (sensitivity of 58% and specificity of 78%); results similar to what we report here (27). Other authors have found different and higher values ​​of ***SRN/M*** with ***C/O*** of 3.59 (reporting a sensitivity, specificity, and AUC of 100%, 86.4%, and 0.969, respectively) (11). Possibly the ***value B*** chosen by them was different or certainly softer than the TN so that the ratio is very high. Görgülü et al. also compared ***SRN/T*** vs. ***SRN/M*** and found that the values ​​were significantly successful in differentiating benign from malignant histopathological types (p< 0.001 for both) and reported an ***SRN/M*** with a ***C/O*** of 5.75 (sensitivity of 100%, specificity of 96.3%, and AUC of 0.996), one of the highest reported in the literature (11). When AUC was compared for both methods, the difference was 0.0265 and was statistically significant (p= 0.046) and the diagnostic accuracy of the ***SRN/M*** was superior to that of the ***SRN/T*** (11), this differs from our results. We consider that the variability of the ***B value*** chosen as a sample in the SCM and the similarity of some areas of the muscle tissue with the TN of our sample are responsible for finding a lower ***SR*** than that published. Additionally, imaging acquisition in longitudinal slices and the limited space in the SCM that is included within the ***SE*** measurement box might also be responsible for our results; another factor to consider is that there is no consensus regarding the area to select and how the ***value B*** is chosen in the SCM or any other nearby muscle. We recommend using the ***value A*** of the TN and the ***SRN/T*** for all of the above.

Regarding quantitative TEs, a meta-analysis of 15 ***SWE*** studies, including 1,867 TNs, showed that the sensitivity and specificity of ***SWE*** was 84.3% and 88.4%, respectively (28). Several meta-analyses of the diagnostic accuracy of thyroid ***SWE*** have been performed with divergent results (14), thus the pooled sensitivity and specificity found in some studies seems encouraging, but the clinical usefulness of these analyzes is questionable since several technologies were pooled (***SWE, pSWE, ARFI***), the patient cohorts were very heterogeneous and there were several different ***C/Os*** applied. For this reason, we analyzed each test separately and present the results of each of the Elastographies with different units of measurement, ***C/O*** and individualized diagnostic tests (**Table 1**). Our reported values are within the ranges published by other authors using different equipment brands, especially regarding the most commonly used measurements, such as the ***SWE Emean*** that had a ***C/O*** of 47.5. The study published by Szczepanek-Parulska found values ​​very similar to these, with an average value of 49 kPa (sensitivity: 86% and specificity: 81%) (29). In comparison, ***SWE E max*** has a ***C/O*** in our study of 115 kPa (sensitivity of 79.5%, specificity of 61.6%, PPV of 72.1%, and NPV of 70.4%), whereas some studies report values ​​of 94 kPa (sensitivity: 46%, specificity: 86%) (16), and an updated meta-analysis showcases multiple cut-off values, lower than what was found here, and is unable to reach a consensus on an specific ***C/O***; as there is so much variability in the literature, the tendency in the future will be to use ***Emean*** values ​​which show less variability. Finally, when looking at m/s, we report higher ***C/O*** for ***Emean***, ***Emax*** and ***average pSWE*** (**Table 1**) than what is usually reported in the literature; for instance, publications such as the one by Kyriakidou et al., reported a lower ***C/O*** of 2.65 m/s (***Emean***) with a sensitivity of 73%, specificity of 67% and NPV of 94% (30), and another investigation identified a ***C/O*** for ***Emax*** of 3.54, with a sensitivity, specificity, PPV, and NPV of 79.2%, 71.5%, 26.7%, and 96.3%, respectively (31). One of the factors that can explain these differences is that we measured the complete contour of the TN and other authors only use the most solid regions of the nodule or the isolated solid regions in the case of a mixed TN. We have not found significant use for the value of ***Emin*** with ***SWE***; however, its values ​​are recorded in **Table 1** for future comparison.

With regards to the available literature on ***TE***, it is important to note that Zhang et al, using the same make and model of equipment as ours, published ***C/Os*** using the shear wave G modulus, reporting a Gmax, Gmean and GsD of 15.82 kPa (Sensitivity 79%, Specificity 79%), 6.715 kPa (Sensitivity 86%, Specificity 68%) and 2.00 kPa (Sensitivity 78%, Specificity 64%), respectively (10). The value of the G-mode elastography is three times less than the value of the ***TE*** Young’s modulus (E) and there are not many publications using this type of ***TE***, so it’s difficult to directly compare our results to these. Additionally, a meta-analysis that included only ***SWE*** studies, reported a suboptimal performance of the method as reflected by a sensitivity and specificity of 66% and 78%, respectively (14).

Finally, when assessing ***TE*** against cytopathology (Bethesda) and commonly used prediction scales such ACR TI-RADS and our own scale (AS), we found good correlation between them as shown in **Table 7**. Our recommendation, which was already widely discussed in previous publications (23, 24), is that two prediction classifications should be used. In our experience, when reporting ACR TI-RADS and AS together, classification of the TNs becomes easier; this has been supported by commentaries of fellow clinicians (23, 24). Furthermore, if we add ***TE*** values to these analyses, as an additional measure of malignancy prediction, physicians would be able to properly select potentially malignant TN that should have FNAB, microcarcinomas that should have active surveillance, or potentially benign TN that should not be punctured, thus decreasing the rate of unnecessary procedures or expenditure (7). In reality, there is still a long way to go before standardizing the values ​​between the different brands, but possibly in a future consensus, such values of ***TE*** will be used in addition to well-known predictors such as ACR TI-RADS, ATA, EURO TI-RADS, or K TI-RADS (32) and not just as an isolated tool. We believe that if ***TE*** measurements are combined with prediction tools such as TI-RADS, greater statistical weight and confidence will strengthen the prediction value of the tool, even more when correlated with commonly used sonographic signs (solid, hypoechoic, microcalcifications, height greater than width, jagged edges, etc). It’s likely that future updates of TI-RADS will have quantitative standardized values ​​of ***TE***. The usefulness of such combinations is shown in the literature and report promising results when combining ultrasound and ***TE***, reaching a sensitivity and NPV of 97% (33). On the contrary, other authors report that the diagnostic accuracy of the specificity and PPV were inferior to conventional ultrasound by itself (28), or that neither TE alone nor in combination with US showed better performance in diagnosing thyroid cancer (34). We disagree with these latter conclusions, as mentioned before, in our experience and according to the results of past publications (23, 24), ***TE*** and ultrasound both individually and together present reliable results that correlate well with Bethesda, histopathology and prediction scales (such as ACR TI-RADS or AS).

### Study limitations

Our reported results and ***C/O*** need to be confirmed in the future with a larger number of cases that will result in the strengthening of the statistical tests; we are actively collecting new cases for a future update. Also, our results should be reproduced in countries from other continents so that the population studied will be genetically different from ours (with different risk factors and profiles), in order to confirm the validity of our results. In any case, we believe that especially for Ibero-America, Latin America, and the Caribbean, the values we ​​found are applicable due to similarities in population; however, this should also be confirmed.

In our study when using the ***SE*** we could not establish an ***SRN/M*** consistent with what has been published. We argue that this may be due to multiple factors such as the type of comparative value of the tissue chosen, the type of acquisition plane, the varying size of the ***value B*** in mm, and to the area of the SCM that will always differ and will be impossible to standardize, which is why we do not recommend it. We did not report the ROC curves due to the low precision of the AUC and we give priority to the other results of the diagnostic tests.

## Conclusions

The diagnostic tests carried out for ***SWE***, ***pSWE***, ***Value A*** of the ***SE***, and ***Nodule/Tissue Strain Ratio*** had very good results and few significant differences between them; the type of elastography, its measurement mode and the units in kPa or m/s can be used according to the preferences of each radiologist, however, we do not recommend to use ***Emin*** values ​​with ***SWE***. The MIQR recommended is less than 15% (kPa) and 8% (m/s) and the recommended depth for ***pSWE*** is 1.2 to 1.5 cm. Statistical tests were promising when comparing the different elastographies with Bethesda, ACR TI-RADS and Alpha Score.

## Data availability statement

The raw data supporting the conclusions of this article will be made available by the authors, without undue reservation.

## Ethics statement

The studies involving human participants were reviewed and approved by Alpha Imagen Institutional Review Board. Written informed consent for participation was not required for this study in accordance with the national legislation and the institutional requirements.

## Author contributions

GM, AM, MU, JO, AG and JL-R contributed to the conception, design and execution of the study. JO contributed with the statistical analysis. GM supervised and executed all imaging analysis. Imaging processing and analysis was performed by AG with support of MU. All authors contributed to the article and approved the submitted version.
